# Animal and human mucosal tissue models to study HIV biomedical interventions: can we predict success?

**DOI:** 10.7448/IAS.18.1.20301

**Published:** 2015-11-02

**Authors:** Charlene S Dezzutti

**Affiliations:** 1 Department of Obstetrics, Gynecology, and Reproductive Sciences, University of Pittsburgh School of Medicine Pittsburgh, PA, USA; 2 Magee-Womens Research Institute, Pittsburgh, PA, USA

**Keywords:** HIV prevention, pre-exposure prophylaxis, microbicide, humanized mouse, non-human primate, macaque, mucosal tissue, *ex vivo* challenge

## Abstract

**Introduction:**

Preclinical testing plays an integral role in the development of HIV prevention modalities. Several models are used including humanized mice, non-human primates and human mucosal tissue cultures.

**Discussion:**

Pharmaceutical development traditionally uses preclinical models to evaluate product safety. The HIV prevention field has extended this paradigm to include models of efficacy, encompassing humanized mice, non-human primates (typically Asian macaques) and human mucosal tissue (such as cervical and colorectal). As our understanding of the biology of HIV transmission improves and includes the influence of human behaviour/biology and co-pathogens, these models have evolved as well to address more complex questions. These three models have demonstrated the effectiveness of systemic (oral) and topical use of antiretroviral drugs. Importantly, pharmacokinetic/pharmacodynamic relationships are being developed and linked to information gathered from human clinical trials. The models are incorporating co-pathogens (bacterial and viral) and the effects of coitus (mucosal fluids) on drug distribution and efficacy. Humanized mice are being tailored in their immune reconstitution to better represent humans. Importantly, human mucosal tissue cultures are now being used in early clinical trials to provide information on product efficacy to more accurately characterize efficacious products to advance to larger clinical trials. While all of these models have made advancements in product development, each has limitations and the data need to be interpreted by keeping these limitations in mind.

**Conclusions:**

Development and refinement of each of these models has been an iterative process and linkages to data generated among each of them and from human clinical trials are needed to determine their reliability. Preclinical testing has evolved from simply identifying products that demonstrate efficacy prior to clinical trials to defining essential pharmacokinetic/pharmacodynamic relationships under a variety of conditions and has the potential to improve product selection prior to the initiation of large-scale human clinical trials. The goal is to provide researchers with ample information to make conversant decisions that guide optimized and efficient product development.

## Introduction

Since the identification of HIV as the causative agent of acquired immunodeficiency syndrome (AIDS), advances have been made in the treatment and care of HIV-infected persons with drug cocktails that extend lives. Biomedical interventions for prevention, such as medical male circumcision, treatment for prevention, pre-exposure prophylaxis (PrEP), microbicides and vaccines have been implemented or are being developed/tested with the goal of creating an AIDS-free generation [1]. Many of the advances in HIV biomedical interventions have depended upon preclinical testing to define efficacy, prior to use in humans. Early preclinical testing relied solely on *in vitro* assays typically using primary immune cells or cell lines, which provided limited information on the activity of the drug. The clinical trials undertaken in the 1990s and early 2000s evaluating these products failed to show comparable efficacy to the preclinical testing in most instances. Through the decades, advances in preclinical testing were made to include the use of animals such as “humanized” mice and non-human primates as well as human mucosal tissue cultures. Preclinical models provide researchers with the ability to perform work that would be unethical in humans, such as exposure to virus and other pathogens, high doses of experimental drugs and intensive tissue sampling. While advances in these models are ongoing, there is no one comprehensive model; each provides important information, but limitations exist. Can these limitations be mitigated to more effectively inform product development? This review provides an overview of the humanized mouse, non-human primate and human mucosal tissue models used primarily for evaluating PrEP and microbicide efficacy results.

## Discussion

### Humanized mouse models

Attempts to use mice, rats and rabbits to study HIV pathogenesis and therapeutic interventions began soon after the isolation of HIV, since these animals were already a cornerstone of basic science research efforts. It soon became apparent that these animals could not be infected with HIV [2]. Human co-factors were required for infection, thus leading to the identification of the CD4 receptor [3] and the chemokine co-receptors [4] as critical for HIV entry into human cells. Even with mice engineered to express human CD4 and co-receptors, blocks in HIV replication and processing were identified [5] suggesting that mice needed to be “humanized” (i.e. engrafted with human immune cells) for further development of this model. In the late 1980s, researchers began to manipulate immunosuppressed mice strains such as the severe-combined immunodeficient (SCID) mice. These mice have a genetic mutation that results in a lack of functional T and B cells, but they do have functional NK cells. Using SCID mice, several human-SCID (hu-SCID) models were developed by administering human peripheral blood mononuclear cells/lymphoid tissue [6–8]. These early attempts resulted in mice with limited human immune cell repopulation, including a lack of human cells in the genital tract, with mice eventually succumbing to graft-versus-host disease. However, using hu-SCID mice repopulated with human peripheral blood mononuclear cells, vaginal application of non-nucleoside reverse transcriptase inhibitors (NNRTIs) [9,10] protected against cell-associated HIV in the presence of semen. These early studies demonstrated that vaginal application of antiretrovirals can prevent cell-associated HIV from infecting the mice and encouraged further development of this model. Refinements of the humanized mouse model over the past decade were done using non-obese diabetic (NOD)-SCID mice or recombination-activating gene (RAG) knock-out mice (RAG^−/−^) in which the IL-2 receptor common gamma chain gene knock-out was introduced, thus allowing these mice to better accept the transplanted cells/tissue and demonstrate immune repopulation throughout the animal, including the mucosa [11–14]. RAG-hu mice have been used to evaluate neutralizing anti-HIV antibodies passively transferred or continuously produced either by i) implantation of the antibody-producing tumour cells or ii) the incorporation of the antibody gene into adenoviral vectors administered to the mice [15,16]. These mice were protected from parenteral HIV challenge. Topical application of a gel delivering a broadly neutralizing monoclonal antibody, VRC01, also protected RAG-hu mice from vaginal HIV challenge [17]. Other mice have been surgically implanted with foetal thymus and liver, sub-lethally irradiated and then transplanted with autologous CD34+ stem cells obtained from the foetal liver; these mice are termed bone marrow-liver-thymus (BLT) mice [14]. BLT mice have been used extensively to evaluate topical and oral administration of several antiretroviral drugs for protection against vaginal and rectal HIV challenge [18–21]. The work by Denton *et al*. [20] demonstrated protection of BLT mice using a dosing regimen of vaginally administered 1% tenofovir similar to that used in the CAPRISA 004 clinical trial, which demonstrated a 39% reduction in HIV acquisition in women using tenofovir 1% gel peri-coitally [22]. Novel HIV prevention approaches have also been evaluated using the BLT mouse model, such as vaginal administration of interfering RNA molecules against the host proteins CD4 and CCR5 [23,24]. In both cases, mice were protected from vaginal HIV challenge. Collectively, the humanized mouse models have provided additional evidence that antiretroviral-based interventions prevent vaginal, rectal and parenteral acquisition of HIV.

Linking drug activity to drug concentration is important for developing pharmacokinetic/pharmacodynamic (PK/PD) models. PK/PD models allow the estimation of how much drug is needed to be efficacious within those models. Until recently, well-defined PK studies in mice had not been done. In RAG-hu mice administered drugs orally, tenofovir concentrations peaked by two hours in all matrices tested with drug exposure (area under the curve, AUC) in vaginal (14.9 µg×h/g) and rectal (1,000 µg×h/g) tissue exceeding plasma (11 µg×h/ml). Maraviroc peaked by four hours in all matrices with the AUC in vaginal (2.4 µg×h/g) and rectal (32.7 µg×h/g) tissue exceeding plasma (0.76 µg×h/ml) [25]. These data are consistent with human PK studies [26,27]. Additional work is needed to model PK/PD correlates of protection in these mice to define variables in the model such as potential differences in protein binding of antiretrovirals in mouse serum compared to human serum and differences in drug metabolism/clearance (Table 1).

**Table 1:** Humanized mouse models

Pros	Cons
Engraftment of human hematopoietic tissue to create a “human” immune system.	Deficiencies in all the human immune cell types.
Infect with HIV, including “transmitter/founder” viruses.	Lack human innate immunity.
Mice will succumb to “wasting” disease similar to humans.	Inability to evaluate non-hematopoietic HIV interactions.
Mice being created to more accurately reflect human innate/adaptive immunity.	Estrus cycle/endocrine system different.
Microbiome transplants being done.	Restrictions on microbiome.
Pharmacokinetic studies being initiated.	Pharmacokinetics may be affected by mouse serum-binding antiretroviral drugs differently than humans.
	Pharmacogenomics are different from humans.
	Mice take several months to be created *de novo* and kept in controlled environment; expensive.
	Penile challenge has not been attempted.

Despite the success of murine models to define product efficacy, there are several areas that can be exploited to improve humanized mice for use in HIV prevention research, including i) development of a human innate immune system, ii) human microbiome transplantation and iii) repopulation of cells from different origins (e.g. epithelial sheets). All humanized mice lack human innate immunity (Table 1). This includes the accompanying cross-talk between epithelial and immune cells. With the confounding of endogenous mouse innate immunity, it is difficult to distinguish human from mouse responses to pathogens. With the advent of new technologies that can modulate host genes (such as CRISPR and TALEN [28]), NOD-SCID mice are being created with deficiencies in their MHC class II, toll-like receptors and interferon type 1 [29]. These new generations of mice should allow the engrafted cells to establish themselves better and provide a more accurate representation of HIV infection and responses to new HIV preventatives. Further, it is anticipated that these mice would accept and respond to human microbiome transplantation. There is a greater appreciation that the microbiome influences host immune and disease development. Thus, human faecal transplants are being done to further humanize the mice [30]. Less work has been done to humanize the mouse vagina. Unlike the gastrointestinal microbiome, the vaginal microbiome has been extensively studied in relation to vaginal health and pregnancy outcomes as opposed to modulating immune development [31]. Shifts from a *Lactobacillus*-dominant flora are associated with bacterial vaginosis, which results in a higher vaginal pH and increased susceptibility to sexually transmitted diseases, including HIV along with increased risk of preterm birth. The mouse vagina does not have a *Lactobacillus*-dominant flora and has a neutral pH. Attempts have been made to colonize the mouse vagina with some *Lactobacillus* species [32], but they have not used *Lactobacillus crispatus*, which is associated with human vaginal homeostasis [33]. Finally, the engraftment of other cell types could lead to the development of co-infection models. For example, aside from HIV, BLT mice have been infected with hepatitis viruses, herpes viruses and other pathogens to evaluate pathogenesis and therapeutic drugs [29]. The inclusion of other tissue-specific engraftments such as using vaginal or ectocervical epithelial sheets would allow the testing of *Neisseria gonorrhoea* or *Chlamydia trachomatis*, which require epithelial cells for their replication cycles.

While advances in the humanized mouse model are being made, significant drawbacks in their widespread use have been the need for veterinary expertise for their creation, access to human foetal tissues and facilities to house germ-free animals, which contribute to the expense in development and maintenance of these mice. Experiments utilizing humanized mice can be quite expensive, limiting the number and type of questions that can be addressed.

### Non-human primate models

Simian immunodeficiency virus (SIV) was isolated from an Asian non-human primate lymphoma in 1985 [34,35] shortly after the identification of HIV as the causative agent of AIDS. Because non-human primates are physiologically and immunologically similar to humans, researchers began to use Asian macaques [rhesus (Indian and Chinese) (Macaca mulatta), pig-tailed (Macaca nemestrina) and cynomolgus (Macaca fascicularis)] to characterize the early transmission events and opportunities for intervention [36–38]. SIV is susceptible to nucleoside/nucleotide reverse transcriptase inhibitors (NRTIs), protease inhibitors and integrase inhibitors, but not NNRTIs, which are all active against HIV [39–41]. This gave researchers a useful model to evaluate some of the HIV therapeutics. In a seminal paper, Tsai *et al*. [40] demonstrated that pre-dosing up to 48 hours with the NRTI tenofovir could prevent infection from a high parenteral SIV challenge. This success opened the door to evaluating antiretroviral drugs as HIV preventatives, not just therapeutics. Tenofovir was also effective at preventing oral SIV transmission to neonates, ushering in a new intervention for the prevention of mother-to-child transmission [42]. To expand the utility of this model, concurrent work resulted in HIV/SIV chimeras – SHIVs. The first SHIV incorporated an HIV envelope and was used to successfully infect the macaques [43]. The creation of a reverse transcriptase (RT)-SHIV by replacing the SIV RT region with one from HIV also demonstrated successful infection of macaques with virus susceptible to HIV-specific NNRTIs [44]. Using these chimeric viruses, topically applied antiretroviral drugs, neutralizing antibodies and entry inhibitors prevented a single, high dose of SHIV from infecting the macaques [45,46]. For these studies, a high dose of SHIV was used, often with progesterone pretreatment (which thins the macaque vaginal epithelium) [47], to ensure the untreated macaques became infected so efficacy could be determined. Because such a high dose of SHIV was required to reproducibly infect the control animals, there was concern the potential efficacy of the drug would be overwhelmed by the non-physiologic challenge. With interest to recapitulate human exposure, a repeat, low-dose mucosal exposure to R5-SHIV (SIV containing a CCR5-using HIV envelope) was developed [48]. The number of viral particles in the low-dose challenge approximated those recovered from semen during acute HIV infection [49] and might provide a more accurate determination of product efficacy. Protection by oral antiretroviral drugs and topical gels containing antiretroviral drugs and entry inhibitors applied vaginally and rectally was demonstrated using the repeat, low-dose SHIV challenge [45]. A high-dose [50] and low-dose [51,52] penile SIV challenge has been developed in macaques as well. While antiretroviral-based prevention has not been evaluated, the penile challenge model was used to demonstrate increased susceptibility to low-dose SIV infection after vaccination with an adenovirus type 5-based SIV gag/pol/nef vaccine [51]. These results were consistent with the findings from the STEP trial, in which the adenovirus type 5 seropositive vaccinees were twice as likely to seroconvert to HIV as the placebo vaccinees [53]. Whether the single, high-dose challenge or repeat, low-dose challenge is the more rigorous way to evaluate the effectiveness of an HIV prevention product remains to be determined (Table 2).

**Table 2:** Non-human primate models

Pros	Cons
Biologically similar to humans.	Require SIV or SHIV; cannot use HIV.
Recapitulate disease pathogenesis.	Innate immunity/host factors different than humans.
SHIVs developed to respond to antiretroviral therapy.	Most species are seasonal breeders, lacking menstrual cycle effects that occurs in humans.
Model HIV transmission events.	Treat with DMPA for reliable infection in single, high-dose model.
Evaluation of sustained vaginal delivery devices.	High-dose/repeat low-dose challenge models.
Susceptible to other sexually transmitted diseases.	Pharmacogenomics similar, but not identical to humans.
Establish pharmacokinetic/pharmacodynamic relationships.	Microbiome different from humans.
	Expensive and availability can be limited.

SIV, simian immunodeficiency virus; SHIV, SIV/HIV hybrid virus; DMPA, depot medroxyprogesterone acetate.

The SHIV/macaque models have allowed researchers to investigate PK/PD relationships of antiretroviral drugs and the timing of drug delivery in relation to viral exposure, which helps to inform the dosage of drugs needed to prevent infection. Because of difficulties with daily oral dosing in macaques, subcutaneous dosing was used for drug administration. Using this strategy, tenofovir disoproxil fumarate with emtricitabine (Truvada™) completely protected against a rectal SHIV challenge as compared to emtricitabine alone, which showed partial protection [54]. Peri-coital dosing, or intermittent PrEP, consisting of tenofovir disoproxil fumarate with emtricitabine protected macaques against rectally administered SHIV as well as daily dosing, thus suggesting high drug levels around high-risk exposure was sufficient for protection [55]. These data were consistent with a human clinical trial, iPrEx, where intermittent use of Truvada (approximately four doses per week) was shown to be very effective against HIV acquisition in a group of high-risk men who have sex with men [56].

Expanding on the non-human primate PK model, Nuttall *et al*. [57] showed topical vaginal administration of tenofovir gel resulted in detectable levels of tenofovir in rectal secretions; conversely rectal administration resulted in detectable levels of tenofovir in vaginal secretions in macaques. These data were confirmed by women applying tenofovir gel vaginally, showing detectable tenofovir levels in their rectal secretions [27]. However, determining whether drug levels in the alternate mucosal compartment are sufficient to prevent SIV/SHIV challenge has yet to be done. This is a critical next step for topical drug administration – multi-compartment protection – as heterosexual couples have reported sequencing between vaginal and rectal intercourse during the same sex act [58]. It is interesting to note that in the non-human primate studies mentioned above and in human clinical trials [59], levels of many antiretroviral drugs are higher in rectal tissue as compared to female genital tissue after oral administration [60]. It remains to be determined if oral PrEP results in lower efficacy for heterosexual women engaging in receptive vaginal intercourse as compared to persons engaging in receptive anal intercourse. Recently, oral administration of maraviroc, a CCR5 agonist, in macaques did not protect against rectal SHIV challenge despite high levels of drug in rectal tissue and fluids [61]. Lack of protection may have been due to increased dissociation of maraviroc from the macaque CCR5 as compared to the human CCR5 [62]. This study highlights the differences in pharmacogenetics between non-human primates and humans (Table 2). Of interest, topical administration may circumvent the dissociation of maraviroc binding CCR5 in the macaques, as three studies showed protection against vaginal and rectal SHIV challenge using topical gels and vaginal rings [63–65]. Topical administration of drug results in several log_10_ more drug locally than can be achieved through oral [27] or likely injectable dosing, which suggests that topical dosing may be advantageous especially for vaginal use.

Similar to the humanized mouse models, differences exist between humans and non-human primates (Table 2). Innate immunity that controls SIV infection in the macaque is different than the innate immunity that controls HIV in humans [66]. For example, macaques do not support HIV-1 infection; HIV-1 replication is blocked before the reverse transcription step. This blockage appears to be due to TRIM5α, a member of the tripartite motif (TRIM) family of proteins, which binds the viral capsid [67]. Human TRIM5α does not associate with the capsid as well as the macaque TRIM5α, which binds it tightly, interfering with the viral reverse transcription processing.

While pig-tailed macaques have menstrual cycles similar to humans, rhesus and cynomolgus macaques are seasonal breeders, suggesting that pig-tailed macaques should be used for evaluating vaginal products and rhesus and cynomolgus macaques should be used for evaluating rectal products. Taking advantage of the pig-tailed macaque model, macaques were evaluated for the “timing” of SHIV acquisition [37]. Almost 90% of the macaques had detectable infection during the follicular phase, leading to speculation that the actual time of infection occurred about a week previous during the luteal phase with accompanying high levels of progesterone. Although immunological changes occur after ovulation in the luteal phase for reproductive success [68], it remains unclear if women are more susceptible to HIV (or other pathogen) infection during this time, as no detailed analysis has been done.

Another important difference between humans and non-human primates is with regard to the vaginal microbiome. Similar to the mouse, the macaque vagina has a neutral pH, with lactobacilli species comprising a minority of the microbiome. The macaque vaginal microbiota is polymicrobial, exhibiting high levels of sialidase activity that resembles the microbiota/enzyme activity of women with bacterial vaginosis [69]. The impact this may have on prevention interventions and SIV/SHIV acquisition is not known. Colonization of *Lactobacillus jensenii* modified to secrete an antiviral protein, cyanovirin-N, demonstrated a reduction of vaginal pH that correlated with higher bacterial colonization levels and reduced levels of some proinflammatory cytokines [70]. Most of the macaques colonized by the *L. jensenii* expressing cyanovirin-N were protected from a repeat, low-dose SHIV vaginal challenge [71]. However, because wild-type *L. jensenii* were not used as a control, it is not clear if the colonization with *L. jensenii*, the cyanovirin-N or both was the mechanism of protection.

To bring human risk factors to the non-human primate model, sexually transmitted pathogen co-infections and the impact of coitus are being evaluated. *C. trachomatis* and *Trichomonas vaginalis* inoculated into pig-tailed macaque vaginas showed similar clinical signs as in humans [72] and the macaques were more susceptible to SHIV infection [73]. A *C. trachomatis* rectal model is also in development [74]. Assessing how these sexually transmitted pathogens affect HIV prevention interventions should help our understanding of the complex PK/PD relationships in conjunction with genital inflammation. Semen is the delivery vehicle for HIV and has been suggested to increase the infectiousness of HIV [75] and reduce the potency of some antiviral drugs [76], thus creating a worst-case scenario for prevention efforts. Defining PK/PD relationships in a non-human primate coital model would address concerns regarding drug potency. Cosgrove-Sweeney and colleagues [77] have developed a coital, pig-tailed macaque model to evaluate topical microbicide safety. They noted genital bruising, shifts in some vaginal flora and a slight increase in vaginal pH in post-coital assessments. However, less than 40% of the post-coital examinations showed evidence of a copulatory plug, so not every mounting resulted in semen deposition. It will be important to define whether immunological changes occur in the non-human primates as have been noted in women after exposure to semen [78,79], as these may affect drug distribution and potency.

With the advances of non-human primates in HIV prevention research, several caveats remain for their widespread use. Non-human primates require veterinary services and secure facilities for housing. Limited availability of non-human primates can restrict experimental design. Macaques are often infected with simian Herpes B virus, which generally remains a latent infection. The animals are asymptomatic during virus reactivation and can transmit virus to their handlers [80], veterinarians and laboratory workers who are scratched, bitten or come in contact with infectious materials such as blood or tissues. Herpes B virus is often fatal to humans and guidelines have been written to minimize exposure and for treatment options if an exposure occurs [81]. Consequently, non-human primate experiments can be quite expensive, limiting the number and type of questions that can be addressed.

### Human mucosal tissue models

Because sexual transmission is the primary mode of HIV infection, use of human mucosal tissue cultures to evaluate drugs and their formulations for potency was a natural model to incorporate into preclinical testing algorithms. Cervical tissue was the first to be used to define the early events of HIV infection and assess the first generation of topical microbicides [82,83]. Since that time, other mucosal tissues have been used, including colorectal, vaginal, tonsil, foreskin and penile. However, the majority of drug evaluation has been done using cervical, colorectal and more recently penile tissues. Use of tissues requires institutional ethics board approval because they are acquired as surgical tissue remainders through local tissue procurement programmes or can be purchased from a company (such as National Disease Research Interchange, www.ndriresource.org/, or Tissue for Research, www.tissueforresearch.com/). While cadaver tissue is available, it has not been used routinely in this context. The tissue is brought to the laboratory where it is set up in two different ways: non-polarized or polarized. Non-polarized tissue is composed of small cubes of tissue retaining the epithelium and lamina propria [83–85]. The tissues are submerged in medium containing HIV with or without drug. This creates a worst-case scenario by allowing virus access to targets in the lamina propria independent of traversing the epithelium. Non-polarized tissues have the advantage of utilizing all of the available tissue as compared to polarized tissues, which use 3 to 5 mm dermal punches and some tissue remains unused. Consequently, many tissue replicates are possible for each treatment condition. Using non-polarized tissue, unformulated drugs have been tested to determine the effective concentrations for HIV [83,84,86–97] as well as HSV2 infection [98,99]. Several entry inhibitors and non-nucleoside and nucleotide RT inhibitors have been tested and show that several log_10_ more drug is needed to inhibit HIV infection of tissue than is needed in traditional *in vitro* assays such as indicator cell lines [84,87,91,92]. These data can be used to define the effective dose that blocks HIV infection in the tissue. Human mucosal tissue models also demonstrated the benefits of drug combinations, which (much like therapy) show an additive effect even in the presence of drug-resistant virus [91,92]. Polarized mucosal tissue has been used primarily to evaluate formulated drugs because they are applied directly to the epithelium. Mucosal tissue is oriented with the apical surface upward and sealed around the sides to maintain a liquid–air separation [82,100,101]. HIV and the formulation are applied to the apical surface, mimicking their delivery in humans. A variety of non-specific entry inhibitors, NNRTIs and NRTIs – alone and in combination – have been incorporated into hydrogel bases and have demonstrated protection against HIV infection [100–108].

To provide persons more options in delivery systems, alternative dosage forms are being developed, including quick dissolve films, tablets, subliming solids and vaginal rings [109]. Efficacy evaluation of solid dosage forms requires a more rigorous approach. Much like the repeat, low-dose challenge model used in non-human primates, where virus is applied multiple times over a period of time, a multi-day challenge has been developed to test the efficacy of products intended for use over extended periods of time, like a vaginal ring. The solid dosage form is placed on the apical surface of cervical tissue and HIV is applied over several days to mimic several high-risk exposures. This model has been used to evaluate ring segments and subliming solids showing protection from the active product, but not a placebo [110].

More recently, human explant tissues have been used to evaluate the effects of hormones and the co-pathogens such as HSV2 on HIV susceptibility. With interest growing in the role of endogenous and exogenous progesterone on HIV susceptibility, a few studies have evaluated mucosal tissue from pre- and post-menopausal women. Rollenhagen and Asin [111] showed cervical tissue from post-menopausal women replicated HIV to higher levels than tissue from pre-menopausal women despite similar levels of proviral copies. Higher replication was associated with more inflammatory mediators secreted from post-menopausal tissue. These data are in contrast to a paper by Saba *et al*. [112], which showed better HIV infection and replication in tissues from pre-menopausal women obtained in their luteal phase, when progestin levels are highest. Poor HIV infection and replication were found in tissues obtained from women during the follicular phase or post-menopause, which were associated with high secreted levels of several chemokines that block HIV infection, namely MIP-1α and RANTES. Additional work to understand how changes in progestin influence HIV replication and response to antiretroviral drugs *ex vivo* is needed. Conversely, co-infection of cervical explants with HSV2 and HIV resulted in more robust HIV infection (more integrated provirus, release of p24) as compared to HIV only [113]. Moreover, when treated with tenofovir, 100-fold more drug was needed to suppress HIV (and HSV2) infection in the co-infected explants as compared to HIV-only-infected explants. HSV2 infection increased the number of activated target cells. Tenofovir may have been metabolized more quickly in the co-infected explants, thus requiring more drug for viral suppression; however, this possibility was not tested. These data show tissue used *ex vivo* responds to external influences and should provide more information on the role of hormones and co-pathogens on HIV prevention modalities.

While mucosal tissue has been useful evaluating new drug entities and formulations, its use has expanded in an innovative manner to an assay termed “*ex vivo* challenge.” Unlike explant cultures, which typically use surgically resected tissue that are exposed to the drug in the laboratory, the *ex vivo* challenge assay obtains biopsies from participants after use of a product for a period of time. The biopsies are brought to the laboratory, where they are exposed to HIV to determine if the product was able to prevent/block infection. The *ex vivo* challenge assay was first used in clinical trials evaluating vaginal gels for safety after rectal application. Participants used the gel for a week and colorectal biopsies were taken, transported to the laboratory and challenged with HIV. In both studies, HIV replication was suppressed in tissue taken from active gel users but not from placebo gel users [114,115], and PK/PD relationships were made between drug levels in the tissue and HIV suppression [116,117]. The *ex vivo* challenge assay has now incorporated cervical and/or vaginal tissue taken after use of study products delivering antiretroviral drugs, including 28-day intravaginal rings or seven daily doses of topical gels or films. Much like the findings from the rectal safety studies, HIV was suppressed in participants using the active products and PK/PD relationships were developed [118,119]. While this assay requires logistical coordination between the clinic and the laboratory, the results will help inform target drug levels needed to block HIV infection *ex vivo*. Because the amount of virus added to this assay is several log_10_ higher than viral titres in semen and a laboratory-adapted virus is used for challenge, how these results translate to effectiveness in humans remains to be determined. Recently, Nicol and colleagues compared PK/PD correlates between drug-treated explants and the *ex vivo* challenge assay [120]. They used explants dosed in the laboratory with tenofovir or maraviroc to predict whether oral dosing would be protective using their *ex vivo* challenge model. The explants predicted <20% would be protected based on the tissue ED_50_ values of 318 µM and 20 µM for tenofovir and maraviroc, respectively. However, the *ex vivo* challenge data resulted in 50% protection after oral dosing. While not completely predictive, this result highlights the importance of comparing these models to develop relational correlates.

As with all models, limitations exist for use of *ex vivo* mucosal tissue (Table 3). Because surgically resected tissue is obtained through the tissue procurement process, there typically is not a regular schedule for receipt of tissues, thus the timing and setup of experiments must accommodate tissue availability. Surgical remainders are from individuals that have undergone planned surgeries and thus have likely received therapy – for example, chemotherapy for cancer or hormonal replacement therapy for gynaecological conditions – which could affect PK and PD responses. Further, surgical remainders are often from an older population, and it was recently shown that HIV replicates to lower levels in these tissues as compared to tissues acquired as biopsies from a younger population [104]. However, responses to microbicide products were virtually the same between surgical resections and flexible sigmoidoscopy biopsies, providing assurance that the use of surgical resections should reflect responses from younger, healthier persons. The capacity to recruit immune cells is lost; consequently responses to pathogens, like HSV2 [113], are from resident immune cells. The mucus and microbiome are washed away, and bacteria and yeast are prevented from growing with the addition of antibiotics to the culture medium. Within 36 to 48 hours of the culture period, the tissue architecture is lost; for cervical tissue, the epithelium blisters off [101] and for colorectal tissue autolysis is evident [85,100]. Thus treatments are typically completed within this early time frame. Understanding these limitations allows experiments to be designed to provide reliable results. The use of biopsy tissue for the *ex vivo* challenge assay eliminates most of these concerns, as tissue is immediately obtained from a younger population after product use and placed into culture.

**Table 3:** Human mucosal tissue models

Pros	Cons
Immune cells in appropriate ratios.	Lack immune cell recruitment/migration.
Infected with HIV and other pathogens.	Lack microbiome.
Responsive to exogenous hormones.	Loss of tissue architecture over time.
Biopsy tissue collected from younger, healthy population during scheduled clinic times.	Surgical resections collected from older population, with clinical reason for surgery.
Establish pharmacokinetic/pharmacodynamic relationships.	Surgically resected tissue collection is opportunistic; restricted on the location of tissue.
Utilize mucosal secretions to deliver HIV to tissue.	
Evaluates human drug dosing for potential product efficacy (*ex vivo* challenge assay).	

## Conclusions

Although preclinical models are representations of human biology and provide critical information on the potential efficacy of many classes of antiretroviral drugs/products, none of the models can fully recapitulate how humans become infected with HIV or how the drug would function in a human. Many prevention products have been evaluated in each of these models and most of them showed protective effects against their respective virus. While advances are being made with each model regarding variables that affect HIV transmission and responses to an intervention – such as host factors (e.g. serum-binding of drugs, innate immunity, pharmacogenomics, metabolomics and microbiome), viral inoculum, hormonal influences, mucosal secretions and co-infections – more work is needed. Cross-validation among these models and to human clinical trial outcomes would be ideal to develop relational PK/PD models, and this work is now ongoing. However, this would likely need to be performed on each drug class (for example, comparing across NRTI and NNRTI classes) until predictability between these drug classes can be assessed. Despite these challenges, the timing, route and dose of viral exposure, as well as adherence to the intervention, are known, allowing these models to be used to answer defined questions. All of the available data from these models should be used to inform stakeholders which drug/dosage form should be considered to move forward to clinical trials in humans.
